# Robotic repair of an incarcerated hepatic ventral incisional hernia in a rural critical access hospital: a case report

**DOI:** 10.1093/jscr/rjag504

**Published:** 2026-06-26

**Authors:** Forrest Bohler, Abigail Chaiyasate, Joshua R Waggener

**Affiliations:** Oakland University William Beaumont School of Medicine, 586 Pioneer Dr., Rochester, MI 48309, United States; University of Michigan College of Literature, Science, and the Arts, 500 South State Street, Ann Arbor, MI 48109, United States; Department of Surgery, Bitterroot Health - Daly Hospital, 1200 Westwood Dr., Hamilton, MT 59840, United States

**Keywords:** hepatic herniation, ventral incisional hernia, robotic repair, hepatic incarceration, rural

## Abstract

A 73-year-old woman with a known epigastric ventral incisional hernia presented to a rural critical access hospital with progressive pain, irreducibility, and abdominal distension. Laboratory evaluation revealed markedly elevated transaminases, and computed tomography demonstrated herniation of the left hepatic lobe through a small abdominal wall defect. Biochemical evidence of hepatic ischemia in this setting is documented in only a small number of prior reports, and operative intervention was pursued urgently given concern for progressive vascular compromise. A robot-assisted transabdominal preperitoneal repair was performed — to our knowledge the first application of robotic surgery to this rare clinical entity — revealing an incarcerated ischemic hepatic segment with a small capsular laceration. The liver was successfully reduced, and resection was avoided. The patient recovered uneventfully. This case highlights a rare presentation of hepatic incarceration and establishes the feasibility of robotic management in a rural setting.

## Introduction

Herniation of hepatic parenchyma through an abdominal wall defect is rare because the liver is normally secured by multiple ligamentous attachments and protected by the rib cage [1]. Most ventral or incisional hernias contain omentum or bowel, and involvement of solid abdominal organs is uncommon [1, 2]. When the liver is present within a hernia sac, it is most often described in diaphragmatic or large incisional defects [1, 3]. Incarceration of hepatic tissue within a small ventral incisional hernia is therefore unusual and may present with biochemical evidence of hepatic injury secondary to vascular congestion or ischemia.

When hepatic herniation does occur through an anterior abdominal wall defect, it has historically been managed via open laparotomy [1–4]. Ischemic compromise of the incarcerated segment, evidenced by biochemical hepatic injury, is documented in only a small number of the cases reported to date [2, 4, 5]. The application of robotic surgery to this rare clinical entity has not previously been described.

## Case report

**Figure 1:**
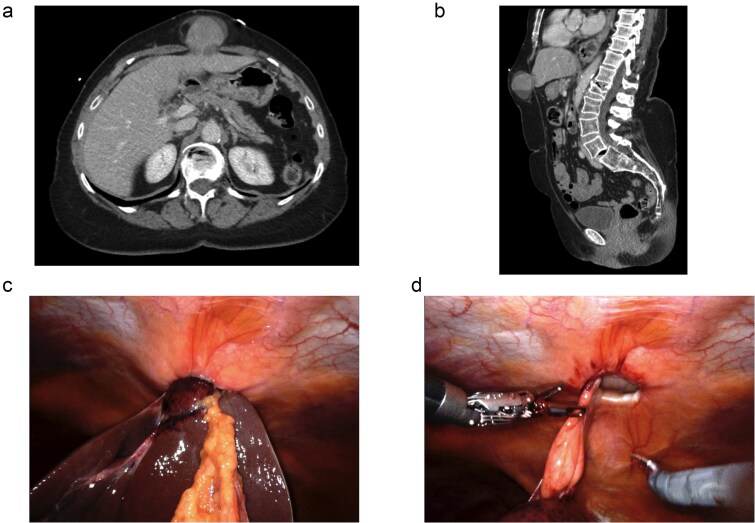
Imaging and intraoperative findings of an incarcerated hepatic ventral incisional hernia. (a) Axial contrast-enhanced CT demonstrating herniation of the inferior aspect of the left hepatic lobe through an anterior abdominal wall defect into the ventral hernia sac. (b) Sagittal CT illustrating protrusion of hepatic parenchyma through the epigastric abdominal wall defect. (c) Intraoperative robotic view demonstrating incarceration of the left hepatic lobe within the ventral hernia defect prior to reduction. (d) Robotic reduction of the incarcerated hepatic segment with closure of the fascial defect during transabdominal preperitoneal ventral hernia repair.

A 73-year-old woman with a known but previously asymptomatic epigastric ventral incisional hernia presented to the emergency department at a critical access hospital in rural Montana with three days of progressively worsening upper abdominal pain and enlargement of the hernia bulge. She reported that the protrusion had become painful, irreducible, and was associated with abdominal distension and new constipation. She denied nausea, vomiting, or symptoms of bowel obstruction. On examination she was haemodynamically stable but had a firm, irreducible epigastric bulge measuring ~6 cm without overlying skin changes. Laboratory evaluation demonstrated mild leukocytosis and marked elevation of liver enzymes, with an aspartate aminotransferase of 305 U/L and alanine aminotransferase of 260 U/L. A contrast-enhanced computed tomography (CT) scan of the abdomen and pelvis revealed a ventral abdominal wall hernia containing the inferior tip of the medial segment of the left hepatic lobe with adjacent free fluid suggesting inflammatory change (Fig. 1a and b). Given concern for incarceration of hepatic parenchyma and evolving hepatic injury, the patient was taken urgently to the operating room.

A robot-assisted laparoscopic ventral incisional hernia repair was performed using a transabdominal preperitoneal approach. Intraoperatively, a 3 × 3 cm fascial defect was identified containing an incarcerated portion of the left hepatic lobe with dark fluid present within the hernia sac (Fig. 1c and d). Gentle laparoscopic manipulation combined with external pressure allowed reduction of the herniated liver. After reduction, the involved hepatic segment appeared initially ischemic and a small ~2 cm capsular laceration was noted on the inferior surface, which was cauterized to achieve hemostasis. The liver was observed during the remainder of the procedure and progressively reperfused, returning to near-normal color, allowing hepatic resection to be avoided. During the preperitoneal dissection a second occult ventral hernia measuring ~2 × 2 cm containing preperitoneal fat was identified several centimeters inferior to the symptomatic defect. Both defects were closed primarily and reinforced with a 10 × 10 cm self-gripping mesh placed in the preperitoneal space. The peritoneum was closed over the mesh and the procedure was completed without complication. Estimated blood loss was minimal.

The patient had an uneventful postoperative course. By postoperative day one she was tolerating a diet, ambulating independently, and her pain was well controlled without narcotic medications. She was discharged home in stable condition the following day. At postoperative follow-up ~10 days later she reported minimal soreness, normal bowel function, no concerns regarding the surgical incisions, and normalization of the previously elevated liver enzymes, consistent with successful reduction and reperfusion of the incarcerated hepatic segment. Examination demonstrated a soft, non-tender abdomen with well-healed incisions.

## Discussion

Herniation of hepatic parenchyma through the anterior abdominal wall is exceptionally rare, with fewer than 20 cases reported in the literature to date. Most have occurred in the setting of incisional hernias following upper abdominal or cardiac surgery, though primary hernias without prior incision have also been described [1, 3]. The majority of reported cases have been managed via open laparotomy, and in at least two instances the incarcerated hepatic segment did not recover following reduction, necessitating partial liver resection [2, 6]. Biochemical evidence of hepatic ischemia in the form of markedly elevated transaminases has been documented in only a small number of reported cases, reflecting the rarity of true vascular compromise in this setting and underscoring the urgency of operative intervention when it occurs [2, 4, 5].

To our knowledge, only one prior case report has described a minimally invasive repair of a ventral incisional hernia containing hepatic parenchyma, using a standard laparoscopic intraperitoneal underlay mesh technique in the elective setting without evidence of incarceration or enzymatic elevation [7]. No prior report has described robotic repair of an incarcerated hepatic hernia of the anterior abdominal wall with threatened ischaemic compromise, nor the use of a robotic platform to facilitate atraumatic reduction and intraoperative monitoring of hepatic reperfusion as an alternative to resection.

The robotic transabdominal preperitoneal approach provided excellent visualization and dexterity, allowing careful manipulation of the incarcerated hepatic segment and atraumatic reduction. The enhanced visualization also facilitated identification of a second occult defect during the preperitoneal dissection, which may have been missed with more limited approaches. Placement of mesh in the preperitoneal plane allowed wide reinforcement of the abdominal wall while maintaining separation between the mesh and intra-abdominal viscera.

This case highlights that complex abdominal wall pathology can be safely managed robotically in rural critical access hospitals when surgeons possess broad operative training and access to modern, minimally invasive platforms. Patients in rural regions frequently face barriers to referral, including long travel distances and delays in accessing tertiary surgical care [8, 9]. In this patient, timely surgical intervention at the presenting hospital allowed prompt reduction of the incarcerated hepatic segment and likely prevented progression to irreversible hepatic injury. This case therefore underscores the importance of maintaining a well-trained rural surgical workforce capable of addressing diverse and occasionally unusual surgical pathologies while utilizing contemporary surgical techniques [10].
